# The Role of Antioxidant Enzymes in the Ovaries

**DOI:** 10.1155/2017/4371714

**Published:** 2017-09-24

**Authors:** Shan Wang, Guolin He, Meng Chen, Tao Zuo, Wenming Xu, Xinghui Liu

**Affiliations:** ^1^Department of Obstetrics and Gynecology, West China Second University Hospital, Sichuan University, Chengdu 610041, China; ^2^Joint Laboratory of Reproductive Medicine, Sichuan University-The Chinese University of Hong Kong (SCU-CUHK), West China Second University Hospital, Sichuan University, Chengdu 610041, China

## Abstract

Proper physiological function of the ovaries is very important for the entire female reproductive system and overall health. Reactive oxygen species (ROS) are generated as by-products during ovarian physiological metabolism, and antioxidants are indicated as factors that can maintain the balance between ROS production and clearance. A disturbance in this balance can induce pathological consequences in oocyte maturation, ovulation, fertilization, implantation, and embryo development, which can ultimately influence pregnancy outcomes. However, our understanding of the molecular and cellular mechanisms underlying these physiological and pathological processes is lacking. This article presents up-to-date findings regarding the effects of antioxidants on the ovaries. An abundance of evidence has confirmed the various significant roles of these antioxidants in the ovaries. Some animal models are discussed in this review to demonstrate the harmful consequences that result from mutation or depletion of antioxidant genes or genes related to antioxidant synthesis. Disruption of antioxidant systems may lead to pathological consequences in women. Antioxidant supplementation is indicated as a possible strategy for treating reproductive disease and infertility by controlling oxidative stress (OS). To confirm this, further investigations are required and more antioxidant therapy in humans has to been performed.

## 1. Background

Reactive oxygen species (ROS) are formed during normal metabolism of oxygen and are produced as by-products of aerobic metabolism. A certain amount of ROS production is necessary for gene expression [1], cell signalling [2, 3], and redox homeostasis. Scavenging antioxidant systems are indispensable for maintaining an adequate amount of ROS. The balance between the generation and elimination of ROS is a key factor required for almost every metabolic function in mammals. Maintenance of this balance is an important constitutive process and has a particular influence on cell proliferation, differentiation, apoptosis, and death [4]. When ROS production overwhelms the scavenging ability of antioxidants, oxidative stress (OS) occurs. Unfortunately, disruption of this balance can easily result from either an increase in the concentration of ROS or a decrease in scavenging ability. Excessive ROS levels are harmful to the human body and can result in accumulation of oxidative damage in distinct subcellular compartments that exert very toxic effects on DNA, proteins, and lipids. ROS-mediated damage can ultimately influence physiological functions, such as cell signalling pathways and redox-sensitive signalling pathways, and lead to pathological conditions [5].

Regarding the female reproductive system, ROS and antioxidants have been recognized as key factors involved in ovarian physiological metabolism. Many studies have investigated the presence of antioxidants and their transcripts in the female reproductive tract [6–8]. Previous studies have reported that the balance between ROS and antioxidants greatly influences the reproductive activities in female mammalian animals, such as endometrial changes in different luteal phases, folliculogenesis, ovulation, fertilization, placental growth, embryogenesis, and implantation [9]. However, under OS conditions, compromised reproduction and fertility may be induced, including impaired ovarian functions, deteriorated oocyte quantity, embryonic development disorders, gynaecological disease, and infertility [10–13]. Thus, antioxidants are critical for maintaining the redox balance in the ovaries to support normal ovarian function. However, their exact molecular mechanisms and roles have not been fully elucidated. Previous studies have primarily focused on ROS functions in the ovaries. Thus, in this context, a systematic understanding of antioxidant expression, regulation, and molecular mechanisms involved in ovarian function is required. Reproductive diseases caused by reduced antioxidant system capacity are also described, and for this reason, future investigations of possible antioxidant supplementation to protect against these diseases are necessary.

## 2. Metabolic Mechanism of ROS and Antioxidants

### 2.1. Reactive Oxygen Species

Reactive oxygen species include radical species, for example, the superoxide anion radical (O_2_^•−^). Although hydrogen peroxide (H_2_O_2_) does not contain unpaired electrons and is therefore not a radical, it is still considered as a form of ROS. The hydroxyl radical (^•^OH) is the most highly reactive and toxic form of oxygen [14]. In addition to ROS, reactive nitrogen species (RNS) have similar effects on cells. RNS include radical species, such as primary nitric oxide (^•^NO) [15].

The redox state of cells is maintained by multiple enzymes and factors. O_2_^•−^ production is normally the initial step of ROS production and propagation, and thus, O_2_^•−^ is considered the precursor of other ROS and functions as a regulator in oxidative chain reactions. After O_2_^•−^ dismutation, H_2_O_2_, which is relatively stable and able to pass through cell membranes, is formed [16]. In the presence of O_2_^•−^, H_2_O_2_ and iron, the Haber-Weiss reaction will occur, which generates ^•^OH [17, 18]. In addition, NO and peroxynitrite (ONOO−) are very important radical species in cells. NO is a well-known key factor for many cellular events and acts as an inhibitor for cell apoptosis and death in a wide range of mammalian cells [19–21]. NO is usually generated from L-arginine via NO synthase (NOS) activity [15] in mitochondria because NOS is located in mitochondria [22, 23]. As a reactive nitrogen intermediate, ONOO− exerts pro-oxidant actions more often than NO itself [24].

### 2.2. Antioxidant Systems

Antioxidant mechanisms exist in all organisms, which enable them to cope with oxidative environments and help cells repair the damage caused by ROS [25]. These mechanisms can be divided into nonenzymatic and enzymatic mechanisms. Enzymatic antioxidants include catalase, SOD, glutathione peroxidase (GPX), glutathione reductase (GR), and glutathione oxidase (GPX). SOD, catalase, and GPX are the three most common enzymatic antioxidants, and they play critical roles in removing the harmful oxygen products produced by superoxide dismutase [26]. Glutathione (GSH) is considered the major representative of nonenzymatic antioxidants present in oocytes and embryos [27]; other types of nonenzymatic antioxidants include vitamin C, vitamin E, selenium, Zn, carotene, and beta-carotene [28].

**Figure 1:**
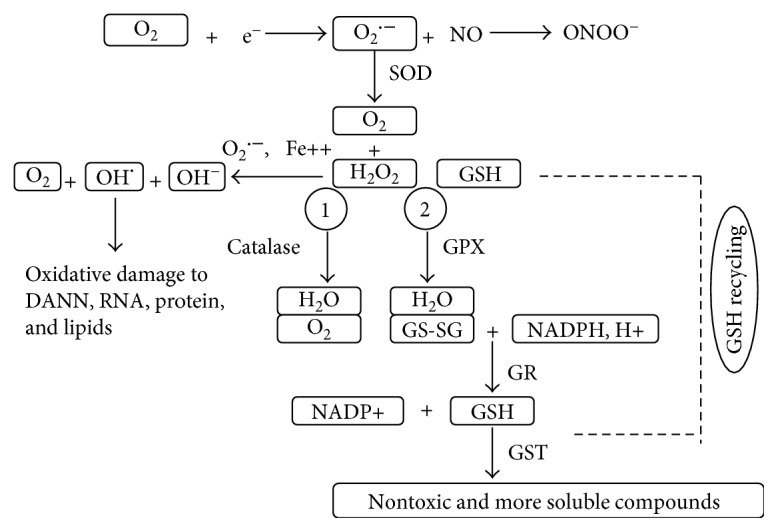
Schematic representation of reactive oxygen species (ROS) generation and important cellular enzymatic antioxidant pathways. The formation of ^•^O_2_ is the initial step in a cascade that results in the formation of other ROS. Mammalian cells contain a variety of antioxidant mechanisms to maintain ROS at a certain concentration. The major antioxidant enzymes include SOD, catalase, GSH, GPX, GR, and GST. These enzymes work together to form a defence system against ROS damage.

The most harmful ROS, O_2_^•−^, is removed by SOD in a dismutation reaction, which is considered the initial and first vital step for regulating intracellular O_2_^•−^ production. The products of this reaction include molecular oxygen (O_2_) and hydrogen peroxide (H_2_O_2_) [16]. To date, three different widely expressed types of SOD have been recognized in organisms. Cu/Zn-SOD (SOD1) is located in cytoplasm and nuclear compartments, while Mn-SOD (SOD2) is expressed in the mitochondria [29]. SOD1 and SOD2 are dimeric and homotetrameric proteins, respectively. EC-SOD (SOD3) is a tetrameric glycoprotein located in the extracellular space [30, 31]. Following the dismutation reaction, hydrogen peroxide can be catalysed by either GPX or catalase into H_2_O. GPX is present in both the mitochondria and cytoplasm [32], while catalase is detected only in peroxisomes [33]. After converting H_2_O_2_ into H_2_O, reduced GSH is oxidized to GSSG (oxidized glutathione) in a peroxidase reaction. Within cells, GSSG is reduced by NADPH. NADPH then regenerates GSH through the enzymatic activity of GR [33]. This entire process is called “GSH recycling,” and it is of fundamental importance for the oxidant scavenging ability of cells. Glutathione transferase (GST) is an enzyme that belongs to a family of multifunctional enzymes. GST plays a vital role in detoxifying reactive metabolites by catalysing their conjugation with GSH. Some of these reactive metabolites are different types of electrophilic alkylating compounds, which are the products generated from OS in macromolecules or biological membranes [34]. GST can transfer the reactive compounds to subcellular sites for further excretion and/or transformation [35].  Figure 1 illustrates a portion of the ROS and antioxidant metabolic pathways.

In addition to the endogenous antioxidants mentioned above, vitamins, for example, vitamin C and vitamin E, among other dietary antioxidants, can also directly scavenge ROS with very high efficiency. Through this scavenging activity, vitamin C and vitamin E provide another major source of protection from ROS-induced damage in cells [19, 36, 37]. Trace elements are also vital antioxidants because they can provide an active site in cells necessary for antioxidants to function properly or participate in the regulation of antioxidant enzymes as cofactors [38, 39]. Polyphenols are ubiquitous in a healthy diet, found in fruits and vegetables, and act as “ROS cleaners” or natural antioxidants. Hence, polyphenols are particularly important for the human body [40].

## 3. A Brief Overview of the Physiological Roles of ROS in the Ovaries

ROS exert both negative and positive effects in mammalian ovaries [41]. From oocyte maturation to fertilization, ROS affect multiple physiological and pathological activities in the ovaries.

Different markers of OS have been examined in cycling ovaries [42, 43]. Macrophages, leukocytes, and cytokines, which are major sources of ROS, are all present in the follicular fluid microenvironment. ROS in the follicular fluid are involved in follicular growth, oocyte maturation, and ovarian steroid biosynthesis [44]. Angiogenesis is a critical process for ovarian folliculogenesis, dominant follicle selection, corpus luteum formation, and embryo formation [45, 46]. Angiogenesis is promoted by oestrogens with some cellular factors, such as VEGF [47]. ROS produced from NADP(H) oxidase were reported to be critical for angiogenesis in vivo and VEGF signalling in vitro [48]. Accordingly, ROS are involved in follicular growth in part by regulating angiogenesis. The development of follicles from the primordial stage to antral follicles is accompanied by a marked increase in the metabolic function of granulosa cells, especially a large increase in cytochrome P450 activity with steroid biosynthesis [49]. Large amounts of ROS are produced during electron transport, indicating that functional granulosa cells are related to the pro-oxidant state in the follicles. When the preovulatory follicle is formed, ovulation occurs under luteinizing hormone (LH) stimulation [50]. A certain amount of ROS is required for ovulation [41], and inhibition of ROS has been confirmed to hinder ovulation [41, 51]. In preovulatory follicles, excessive antioxidants impair LH-stimulated progesterone secretion and ovulation-related gene expression [41]. ROS are induced in preovulatory follicles with oscillation of prostaglandins, cytokines, proteolytic enzymes, and steroids, resulting in blood flow alterations and eventual follicle rupture [52]. The ovulation process is compared with an acute inflammatory reaction because many genes and reagents related to inflammation are induced in the preovulatory follicles by the LH surge during ovulation [53, 54]. ROS function as critical modulators during the inflammatory reactions involved in follicular rupture [55]. With the exception of dominant follicles, which are released for fertilization, the other growing follicles will all undergo apoptosis, and this process is promoted by ROS. In parallel, FSH-induced oestrogen synthesis and upregulation of catalase and GSH in growing follicles counteract the apoptotic process to maintain the balance during normal ovarian function [56]. During the luteal phase, a large amount of progesterone is produced to maintain the early stage of pregnancy. If pregnancy does not occur, the corpus luteum regresses [56]. A rapid reduction in progesterone is required for adequate follicular maturation in the next reproductive cycle. ROS are generated in the corpus luteum and are involved in functional luteolysis. ROS and antioxidants are related to progesterone synthesis in the luteal phase [27]. Steroidogenesis is a major source of ROS production, and progesterone synthesis is restricted in the corpus luteum with ROS [57]. During pregnancy, decreased SOD1 induces an increase in ROS production, resulting in progesterone inhibition, and thus, scavenging of ROS by antioxidants may contribute to the maintenance of luteal cell integrity and extend the life span of the corpus luteum [58].

## 4. Antioxidants in the Ovaries

As described in Background, as the defence system for maintaining the redox balance under physiological conditions, antioxidants have a large influence on reproductive activities [9]. Historically, scientists have emphasized the function of ROS in female reproduction rather than that of antioxidants, and most papers are related to reproductive activities that occur after ovulation, while the follicular growth process is rarely discussed. Thus, we have reviewed antioxidants and their relative roles in almost all ovarian activities. Among all the antioxidants, we chose to discuss the most significant antioxidants, including catalase and SOD, and the antioxidants involved in GSH recycling. Their regulation during the ovarian cycle and follicular maturation are systematically reviewed. In addition, relevant literature is listed and discussed with regard to the roles of antioxidants in oocyte maturation, ovulation, corpus luteum function, and steroidogenesis. The possible regulatory function of gonadotropins on antioxidants is also addressed here.

### 4.1. Catalase

Catalase plays a critical role in ROS metabolism. For this reason, catalase has been intensely studied in recent years. However, very limited evidence has been found regarding catalase regulation in follicular development and differentiation. Catalase is predominantly found in peroxisomes. In the ovaries, catalase was first detected in 1975 by immunohistochemistry [59]. Catalase expression in oocytes is low compared with other cell types in the follicles [60, 61]. In the oocyte nucleus, chromosomal defects such as chromosome misalignment and DNA damage can be induced after inhibition of catalase, and during meiotic maturation in mouse oocytes, catalase has been shown to protect the genome from oxidative damage [60].

Regarding catalase regulation in follicular growth, the activity of catalase in granulosa cells from large follicles has been observed to be several times higher than that in small and medium follicles in various mammals, such as pigs [62], goats [63], and rats [64]. In rat ovarian granulosa and theca cells, increased catalase activity can be observed during ovarian development and luteinisation [65, 66]. Except in folliculogenesis, catalase content has also been found to be distributed throughout different oestrous phases. Singh and Pandey observed that catalase activity in total ovary homogenate was highest in the metestrus phase, declined in the oestrous and pro-oestrous stages, and reached the lowest levels in the diestrus phase [67]. Nevertheless, catalase concentration in follicular fluid is not significantly different among follicles of different sizes [68, 69].

The distribution and oscillation of catalase during different ovarian cycles are suggested to be related to gonadotropin regulation. Gonadotropins such as FSH have well-known functions that are primarily important for follicular maturation, differentiation, and steroidogenesis [70]. Interestingly, catalase activity has been reported to be significantly enhanced through gonadotropin stimulation in different mammals [63, 71–73]. Behl and Pandey further investigated whether catalase and oestradiol activities in ovarian granulosa cells in different follicle stages were related to FSH levels. They found that not only oestradiol secretion but also catalase activity increased after FSH stimulation, and the degree of this increase was greater in large follicles than in medium or small follicles [63]. As it is well known that oestrogen reaches its highest concentration in dominant follicles, the concomitant increase in catalase and oestradiol in response to FSH may suggest a role of catalase in follicle selection and prevention of apoptosis. After ovulation was blocked, catalase activity increases significantly in the entire follicle as well as in the thecal cells [64]. Furthermore, the activity of catalase in both rat and pig ovaries has been shown to be positively correlated with the amount of ferredoxin and cytochrome P450scc, which are two constituents of the steroidogenic electron transport chain [74]. In steroidogenic biogenesis, oxygen free radicals such as superoxide are produced [75–78] and then catalysed by SOD to H_2_O_2_ [16, 79]. Accordingly, catalase acts as a protective factor to neutralize H_2_O_2_ to maintain ROS balance and normal steroid levels. These studies show that catalase contributes to follicular development, the oestrous cycle, and steroidogenic events in the ovaries.

### 4.2. SOD

SOD enzyme families are present in multiple tissues and ovaries of different mammals. The location of SOD in the human body was first determined by Shiotani et al. [80] and Tamate et al. [43] using immunohistochemistry. Neither SOD1 nor SOD2 has been observed in primordial and primary follicles. SOD2 has been detected in secondary follicles, while SOD1 begins to appear in theca cells after the formation of the antral cavity. SOD1 cannot be detected in granulosa cells until follicles enter the dominant follicle stage. High expression levels of both SOD1 and SOD2 have been detected in luteinized granulosa and theca cells.

Biochemically, a peak of collective SOD activity appears during the pro-oestrus phase, which involves the lowest level of superoxide radicals compared with other oestrous stages [81, 82]. Both the amount and activity of the three SOD isoforms in follicular fluid are greater in small and medium follicles than in large antral follicles, and these findings have been assessed and verified in different animals [68, 69, 83, 84]. Interestingly, compared with follicular fluid, no changes in SOD have been observed in granulosa cells with regard to the size of the follicles [83]. Furthermore, high SOD activity in follicular fluid was correlated with low fertilization rates in humans by comparing SOD activity in follicular fluid from patients whose oocytes did not become fertilized with those whose oocytes did become fertilized [85]. According to the above evidence, relatively decreased SOD activity in large follicular fluid is necessary to ensure that ROS levels reach a threshold value that is required for ovulation. However, excessive ROS production in preovulatory follicles may exert harmful effects to oocytes. An oocyte in the preovulatory follicle acquires developmental competence and a very active metabolism, and during this process, a large amount of ROS can be generated; thus, SOD1 is required to neutralize O_2_^•−^ in the cytoplasm of oocytes [86], and therefore, SOD must be maintained at a certain concentration and activity level within the follicles to guarantee a balance between O_2_^•−^ and H_2_O_2_ for normal cellular function [87]. Conclusively, a certain amount of SOD not only ensures a functional concentration of ROS for ovulation but also protects oocytes from OS. After ovulation, SODs are very active in the corpus luteum, because corpus luteum function is related to progesterone levels and ROS. Interestingly, progesterone fluctuation in the luteal phase is positively correlated with SOD1 activity. Reduction in SOD1 during corpus luteum regression is accompanied by increased ROS levels. In contrast to SOD1, SOD2 concentration in the corpus luteum is enhanced in the regression phase to clear the excess ROS produced in mitochondria by cytokines and inflammatory reactions. Thus, SOD1 activity in the corpus luteum is closely correlated with progesterone secretion, while SOD2 is primarily targeted to protect the luteal cells from oxidative damage caused by inflammation [27].

Successful cultivation of cumulus cell-oocyte complexes (COCs) in vitro has made it possible to analyse the function of the SOD isoforms in oocytes. SOD1 and SOD3 are expressed in the oocyte nucleus, and SOD3 is the only SOD isoform that can be detected in the zona pellucida. The level of SOD1 in the oocyte nucleus is enhanced in small and medium-sized follicles [83]. Interestingly, SOD3 can be translocated from cumulus cells into oocytes under certain conditions [88]. This evidence may demonstrate that SOD1 and SOD3 potentially contribute to the protection of DNA or transcription regulation of redox-sensitive genes. Mammalian oocytes contain many a large number of mitochondria, and SOD2, which is specifically localized to the mitochondria and is the principle scavenger of mitochondrial superoxide [89, 90]. The extracellular matrix, which is an important component of COCs, is dynamically regulated, and SOD3 was shown to be one of the critical regulators [91]. When ovulation occurs, there is a surge in ROS production in COCs, and OS is greatly enhanced [44]. Consequently, COCs may stockpile the various SOD isoforms for upcoming events, such as ovulation, fertilization, and early embryonic development.

Matzuk et al. generated SOD1 null mice with reduced fertility as evidenced by a decreased number of preovulatory follicles and corpora lutea; primary and small antral follicles but few corpora lutea were found in these mice under histological analysis [92]. Another group created a copper chaperone for superoxide dismutase (CCS)-null mice that induced marked reductions in SOD1 activity. CCS (−/−) mice showed abnormal development of the antral follicles and no corpus luteum [93]. Furthermore, SOD1 is reportedly involved in antral follicle development. Matzuk et al. also examined SOD2-deficient mice, and they found that SOD2-deficient mice die within three weeks of birth. After the authors completed transplantation of ovaries from the postnatal SOD2-deficient mice to bursa wild-type hosts, all follicle stages were detected, and viable offspring were obtained, which suggest a less important role for SOD2 than for SOD1 in ovarian functions [92].

To investigate SOD regulation in relation to steroids, oestradiol and SOD were measured in the follicular fluid of patients who underwent in vitro fertilization (IVF). Interestingly, researchers found a strong positive correlation between SOD enzyme activity and intrafollicular oestradiol levels, which are related to oocyte quality [94]. In contrast, SOD was shown to have inhibitory effects on oestrogen synthesis by inhibiting FSH-induced aromatase activity in cultured granulosa cells, and this inhibition was found to occur at one or more post-FSH receptor sites in rat granulosa cells in vitro [95]. LH is a gonadotropic hormone secreted from the anterior pituitary gland [96]. An LH peak triggers ovulation, and LH later stimulates the development of the corpus luteum [96]. Kawaguchi et al. found that LH can increase the mRNA and protein levels of SOD1, SOD2, and catalase as well as SOD activity in the bovine corpora lutea. SOD1, SOD2, and catalase mRNA levels varied in different luteal phases and reached the highest expression in the midluteal phase. In addition, the authors suggested that the LH-induced upregulation of antioxidant enzymes increased cell viability and maintained corpus luteum function during the luteal phase [97]. Conversely, corpus luteum-derived SOD2 was found to serve as an LH-release inhibitory factor in sheep [98].

### 4.3. GSH Recycling

GSH is a low molecular weight thiol that is predominantly expressed in mammalian cells. GSH maintains cells in a reduced state and functions as an electron donor for some antioxidant enzymes [99, 100]. GSH is involved in many cellular functions, including cell proliferation, differentiation, and apoptosis [101]. GSH can be synthesized de novo from glutamate, cysteine, and glycine via catalysis by glutamate cysteine ligase and glutathione synthetase. Another predominant enzymatic system for maintaining GSH in most tissues is the reduction of GSSG to GSH via GR with NADPH consumption [99]. GSH can then be oxidized back to GSSG. In the ovary, the strongest GR activity is found in oocytes [102].

Studies of ovarian functions have demonstrated that the GSH concentration in follicular fluid in large follicles is significantly higher than that in small follicles during the luteal phase [68]. Luderer et al. reported that the GSH content in the ovaries increased from the oestrous to metestrus phase compared with the diestrus to pro-oestrus phase in adult rats [103]. Accordingly, the highest GR activity was detected in the metestrus phase [102]. Surprisingly, Lee et al. discovered that ovarian alpha-class GST expression levels were much higher in the pro-oestrus phase than in the diestrus phase in total ovary homogenates [104]. These results suggest that different enzymes involved in GSH recycling are sensitive to the changes that occur during the oestrous cycle, with different regulation mechanisms and various effects among different follicle sizes. However, the regulatory mechanism of these antioxidants during the ovarian cycle and follicular growth is poorly characterized.

According to the literature, GSH increases gamete viability and fertilization. GSH content was reported to be decreased by approximately 10-fold in unfertilized mouse oocytes [105]. In addition, researchers have described GSH contribution to spindle formation in bovine oocytes via depletion of GSH in oocytes; after GSH depletion, the spindle poles became wider, and the spindle area increased significantly [106]. Furthermore, high GSH content in oocytes during follicular development is related to improved development competence of the follicle [107–109]. In addition to GSH, GPX has been reported to play significant roles in gametogenesis and in vitro fertilization. The activity of GPX in the follicular fluid of follicles that were subsequently fertilized was higher than that in fluid from nonfertilized follicles [110]. During the GSH cycle, GR, which cycles GSSG back to GSH, may also play a pivotal role in ovarian function by maintaining GSH at reduced levels [102].

Both in vivo and in vitro studies have shown that the oocyte GSH concentration is modulated by gonadotropin signalling during the preovulatory period. In vivo studies revealed that FSH stimulation enhanced ovarian GSH content [103, 111]. In addition, the GSH content in cumulus cells was gradually increased during oocyte maturation in pigs [112, 113], cattle [114], and horses [115]. One research group has shown that GSH content was increased in porcine oocytes and cumulus cells under FSH treatment in pigs via modulation of glutamate cysteine ligase (GCL) subunit mRNA [116]. GCL is the rate-limiting enzyme in GSH synthesis. In contrast, another research group observed a reduction in GSH and transcript levels encoding GSH in the presence of FSH alone in COCs of cattle [117], but this phenomenon was rescued by combined treatment with bone morphogenetic protein 15 (BMP15) [117], which is an important factor in ovarian maturation [118, 119]. FSH and BMP15 cotreatment promoted development competence in oocytes by distributing metabolism equally throughout the oocyte. FSH promoted glucose metabolism, while BMP15 accelerated glutathione recycling to protect against cellular OS via increased NADPH production [117].

Gene knock-out mice were created to analyse gene function in the ovaries and during fertilization. Ho et al. created Gpx1-null mice and observed that the mice exhibited normal fertility [120]. Deletion of the entire Gpx4 gene can lead to embryonic death [121]. In comparison, deletion of the mitochondrial form [122] or nuclear form [123] has no effect on female fertility. The fertility of mice with an inactivating mutation in the GR gene was also unaffected [124–126]. Nonetheless, these studies did not investigate the effect of these mutations and deletions on ovarian function. C-Glutamyl transpeptidase 1 (Ggt1) is an enzyme that participates in glutathione synthesis. Ggt1-null mice demonstrated growth retardation and a severe female reproductive phenotype, which included no large antral follicles or corpus luteum in the ovaries and a lack of response of the follicles to exogenous gonadotropin stimulation. All female Ggt1-null mice were completely infertile [127–129], but GSH concentrations were not significantly altered in these mice compared with wild-type mice. Consistent with the Ggt1 mutant phenotype, high-performance liquid chromatography (HPLC) analysis of adult ovaries showed that the intracellular cysteine levels were largely reduced, but interestingly, the female reproductive phenotype was completely rescued by cysteine replacement [127]. GCL is composed of a modifier (GCLM) and a catalytic (GCLC) subunit. GCLM-null mice can survive and reproduce, whereas GCLC-null mice die at the early embryonic stage [130–133]. Oocyte GSH concentrations in GCLM-null mice were less than 20% than those in oocytes of GCLM wild-type mice. Additionally, fertility was markedly reduced due to decreased progression to both the pronucleus and blastocyst stages [134].

ROS are believed to be involved in the initiation of apoptosis, as ROS levels increase prior to any other indicator of apoptosis in follicles. After blocking the synthesis of GSH with the inhibitor buthionine sulfoximine (BSO), a statistically significant increase in atretic antral follicles was observed in rat ovaries [135]. Prevention of apoptosis initiation in antral follicles by FSH is commonly accepted [136, 137]. Interestingly, FSH treatment can reportedly stimulate GSH synthesis [138, 139]. The antiapoptotic effect of FSH on granulosa cell apoptosis can be markedly inhibited by blocking the synthesis of GSH with BSO in cultured follicles [138]. In addition to GSH, catalase and SODs can also protect against apoptosis in large antral follicles in rats [140].

## 5. Disturbance of Redox State under Pathological Conditions and Ageing

Many physiological processes can be influenced by OS, which can lead to negative effects or even cause pathological conditions in reproductive systems [10–13, 141]. One explanation for these pathological conditions may be, at least in part, decreased scavenging capability of antioxidants, which can lead to excessive ROS production. It has been suggested that the pathological consequences of decreased antioxidant defence systems include many reproductive diseases, such as polycystic ovarian syndrome (PCOS), endometriosis, and unexplained infertility, as well as complications during pregnancy, such as early miscarriage, abortion, recurrent pregnancy loss, and preeclampsia. Age-related fertility decline is also reported to be related to decreased antioxidant systems [8, 142–148].

PCOS is one of the most common gynaecological diseases of reproductive-aged women. Clinical manifestations of PCOS include menstrual disorders or skin disorders and reduced fertilization rate. PCOS is characterized by ovulation dysfunction, hyperandrogenism, and polycystic ovaries [149]. Mitochondrial dysfunction, accompanied by decreased GSH levels and O_2_ consumption, is found in PCOS patients [150]. Insulin resistance is considered the major aetiology of PCOS. Studies have indicated that antioxidants, including SOD, vitamin C, and vitamin E, are reduced in PCOS patients, leading to an oxidative status that may further cause an inflammatory environment, insulin resistance, and an increase in androgens [151, 152].

Infertility is a disease that is defined as failure to achieve a clinical pregnancy after 12 months or more of regular unprotected sexual intercourse [153]. If the couple has been confirmed infertile without a known cause of infertility after examination, a diagnosis of unexplained infertility is assigned [154]. Approximately 15% of couples are affected by unexplained infertility. Although the pathophysiology is still unclear, evidence has indicated that increased ROS and decreased antioxidants may contribute to unexplained infertility [155–157]. A reduction in antioxidants, including GSH and vitamin E, was reported in patients with idiopathic infertility [158]. Excessive ROS production caused by pathological conditions, environmental changes, or drug therapy may overwhelm antioxidant defence ability and lead to the deterioration of oocyte quality by inducing apoptosis [87, 159–161]. Evidence has further confirmed that ROS-induced granulosa cell degeneration leads to decreased oestrogen levels and compromised oocyte quality and ovulation rate [162]. Chaube et al. revealed that nutrition and growth factors for follicular maturation were affected when granulosa-oocyte communication was reduced under OS, which led to impaired quality of preovulatory follicles [163]. Even after fertilization, excessive ROS production may lead to implantation failure, embryo fragmentation, impaired placentation, and abortion [164]. During pregnancy, the endometrium contributes to the support of embryo development, and this process may be prevented by ROS overproduction [165]. The corpus luteum is critical for maintaining pregnancy in the early stage, and OS may accelerate luteal regression and inhibit steroid production by the corpus luteum [12].

With an increase in reproductive age, the antioxidant levels within the follicular fluid may gradually diminish. Human studies have shown that the levels of catalase and SOD in the follicular fluid of older women were lower than those in younger women, and older women exhibited lower fertilization rates and reduced blastocyst development [166]. Lim et al. showed that the mRNA levels of mitochondrial antioxidants Prdx3 and Txn2 and cytosolic antioxidants Glrx1 and Gstm2 in mouse ovaries were decreased with increased age, which may influence age-related oxidative damage on ovarian function [167]. Thus, we conclude that the ROS scavenging ability of antioxidants is related to fertilization outcomes.

## 6. Possible Antioxidant Therapy against ROS

Antioxidants are helpful for minimizing OS induced by excessive ROS production by clearing free radicals and lowering ROS levels in the human body [13]. Both enzymatic and nonenzymatic antioxidants can be useful for overcoming OS caused by ROS [13]. Antioxidant supplementation has been confirmed to have positive effects on mouse oocyte quality by reducing the harmful effects of OS [168]. Melatonin has frequently been investigated in recent years. Animal studies have shown that melatonin was able to prevent OS-mediated deterioration of oocyte quality in rats [161, 169]. Additionally, melatonin contributes to improved reproductive outcomes by enhancing oocyte quality in humans [170, 171]. Thus, melatonin is a very important naturally produced antioxidant in mammals. In addition to melatonin, resveratrol was shown to protect against the reduction of fertility with reproductive ageing in mice by enhancing the number and quality of oocytes [172]. Studies have shown that women with endometriosis intake lower daily amounts of vitamins A, C [173], and E [173, 174] than other women. Daily supplementation with vitamins C and E for four months was found to reduce the OD marker in those patients. However, it did not improve the fertilization rate [174]. N-Acetyl-cysteine (NAC) has antioxidant properties, as it is able to increase intracellular GSH concentrations and/or directly scavenge free radicals [175, 176]. The pregnancy outcomes of patients with unexplained recurrent pregnancy loss were improved after taking a combination of NAC and folic acid [177]. However, in some gynaecological diseases such as preeclampsia and spontaneous abortion, antioxidant supplementation was found to be ineffective [178–181]. More studies are needed to investigate the effects of antioxidant supplementation as a possible treatment therapy for these patients. Daily intake of fresh green vegetables and fruits, antioxidant-rich legumes, and plant products that contain high levels of antioxidants may be beneficial for reducing OS [182].

## 7. Conclusion

**Figure 2:**
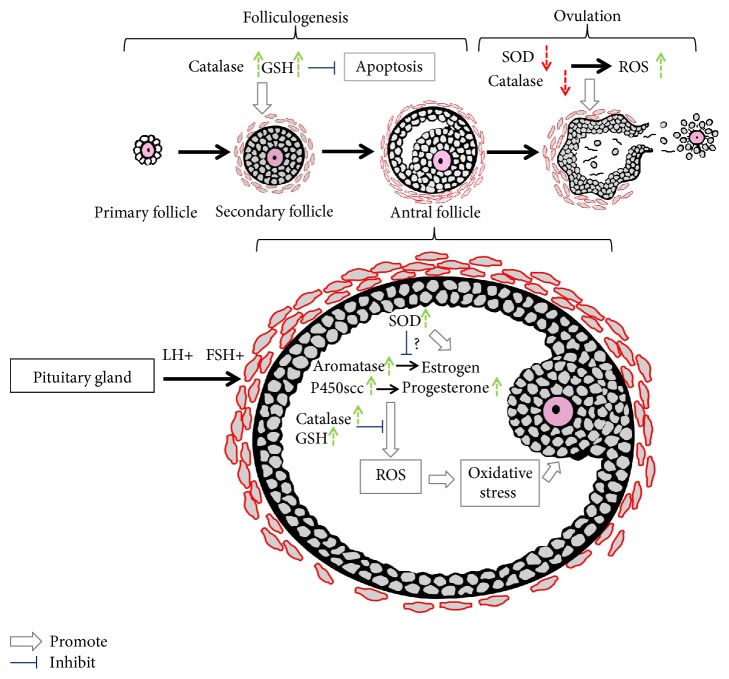
Schematic representation of antioxidant regulation in follicular development. The follicular development process is initiated with primordial follicles to primary follicles, followed by secondary follicles and tertiary follicles. Preovulatory follicles are formed under the stimulation of FSH, and finally, ovulation is triggered by a surge of luteinizing hormone (LH). All these consecutive and synchronized events are accompanied by ROS production and scavenging. Antioxidants are strongly modulated during this process. Catalase and GSH expression in the follicles is enhanced with follicular growth, while SOD activity is reduced in folliculogenesis. SOD was shown to have inhibitory effects on oestrogen synthesis by inhibiting FSH-induced aromatase activity in cultured granulosa cells, while SOD enzyme activity is positively correlated with oestradiol levels in the follicular fluid. A large amount of ROS can be produced during steroidogenesis, especially during the conversion of cholesterol to pregnenolone via cytochrome P450scc. Gonadotropin induces the upregulation of antioxidants such as catalase; GSH in the follicles protects oocytes from oxidative stress generated from physiological metabolic processes, such as steroidogenesis, in the ovary.

Recently, there has been growing interest in the role of antioxidants in female reproductive activities. Antioxidant products and ROS balance have been shown to be closely related to female subfertility or infertility. Substantial evidence has indicated that some physiological processes, from oocyte maturation to fertilization and embryo development, are particularly sensitive to OS. These processes require antioxidants for balanced function. In this review, we thoroughly discussed the expression and regulation of some major antioxidants involved in follicular development, oocyte maturation, ovulation, corpus luteum function, steroidogenesis, and fertilization. Among these reproductive activities, follicular growth, which is reviewed here, has been previously poorly addressed. The general regulation of antioxidants in follicular development is illustrated in Figure 2. However, the molecular mechanisms behind follicular development have not been fully elucidated, and additional evidence for the role of antioxidants in primordial and primary follicles is needed.

The gynaecological diseases presented in this review may result from changes in early ovarian stages due to dysfunction of antioxidant systems. Thus, it is necessary to emphasize the role of antioxidants in the development and survival process of follicles and in follicle responsiveness to gonadotropins as well as in steroidogenesis. Elucidation of the molecular mechanism underlying the involvement of antioxidants in follicular growth, ovarian cycle, oocyte maturation, and ovulation is essential for creating a better understanding of the possible protective effects of antioxidants and for potentially treating female infertility with antioxidant supplementation in vivo.
